# Deficits in latent inhibition induced by estradiol replacement are ameliorated by haloperidol treatment

**DOI:** 10.3389/fnbeh.2013.00136

**Published:** 2013-10-01

**Authors:** Anne Almey, Nada M. Hafez, Arne Hantson, Wayne G. Brake

**Affiliations:** Centre for Studies in Behavioral Neurobiology (CSBN), Department of Psychology, Concordia UniversityMontreal, QC, Canada

**Keywords:** estrogen, typical antipsychotic, selective attention, plasma estradiol, schizophrenia

## Abstract

There are sex differences in the symptomatology of schizophrenia, and in the response to antipsychotic treatments. One hallmark symptom of schizophrenia is a deficit in selective attention. Selective attention can be measured using a latent inhibition (LI) paradigm in humans; LI can be measured in rodents, and is used as an animal model of the selective attention deficits observed in schizophrenia. In the current experiments LI was used to clarify whether selective attention differs between male rats and ovariectomized (OVX) female rats receiving different estradiol (E2) replacement regimens. An additional aim was to determine whether haloperidol’s (HAL) facilitation of LI is enhanced by E2. Males and OVX female rats were trained in a conditioned emotional response LI paradigm. Females received no E2 replacement, a chronic low dose of E2 via silastic capsule, or a high phasic dose of E2 via silastic capsule accompanied by E2 (10 µg/kg subcutaneous (SC)) injections every 4th day. Actual plasma levels of E2 were determined using an enzyme linked immunosorbent assay. Rats were also administered a vehicle treatment, a 0.05 mg/kg, or a 0.1 mg/kg IP injection of HAL. Males and OVX females that did not receive E2 replacement both exhibited LI, but LI was not observed in the low and high E2 replacement groups. HAL restored LI at a lower dose in the females receiving high E2 replacement compared to females receiving low E2 replacement, indicating that E2 replacement facilitates HAL in restoring LI.

## Introduction

There are sex differences in schizophrenia, particularly in the positive symptoms of this disorder, with women developing schizophrenia later in life and exhibiting less severe symptomatology than males (Häfner et al., 1992; Häfner, 2003; Kulkarni et al., 2008). It has been suggested that estrogens are, in part, responsible for these sex differences, reducing the frequency of hospital admissions and diminishing the psychotic symptoms associated with schizophrenia (Kendell et al., 1987; Matevosyan, 2011). Additionally, there is some evidence that estrogen is linked to greater efficacy of antipsychotic treatments, as positive symptoms of schizophrenia are further reduced when antipsychotics are co-administered with estrogen than when administered alone (Akhondzadeh et al., 2003; Seeman, 2004). These studies suggest that estrogens protect against the positive symptoms of schizophrenia and facilitate antipsychotic treatments in ameliorating these positive symptoms. However, it remains unclear whether estrogens also have these effects on the cognitive symptoms of schizophrenia.

One specific cognitive deficit associated with schizophrenia is disrupted selective attention, which is often assessed using a latent inhibition (LI) paradigm (Escobar et al., 2002). With LI, non-reinforced preexposures to a stimulus impair subsequent conditioning, as these preexposures result in reduced allocation of attention to that stimulus (Lubow, 1989). However, individuals with acute schizophrenia and schizoptypy have impaired performance in LI, as repeated non-reinforced preexposures to a stimulus do not retard conditioning to that stimulus (Lubow et al., 2000; Schmidt-Hansen et al., 2009; Kaplan and Lubow, 2011). Individuals with chronic, medicated, schizophrenia do not exhibit disrupted LI, indicating that antipsychotic treatment ameliorates this deficit in selective attention (Gray, 1998). It has been argued that this attentional deficit in schizophrenics, characterized by the processing of irrelevant stimuli, is a critical contributor to the symptomatology of this disease (Brébion et al., 1996; Schmidt-Hansen et al., 2009). Although deficits in selective attention are a hallmark of schizophrenia, few studies have examined whether there are sex differences in this cognitive symptom, and how ovarian hormones may contribute to such differences. Preliminary research in both schizophrenic and non-schizophrenic populations, indicates that males exhibit LI under conditions where females do not (Vol’f et al., 2001; Kaplan and Lubow, 2011). The reason for these sex differences is currently unknown, but it is possible that higher levels of estrogens in females may contribute to this deficit in selective attention. Additionally, antipsychotic treatments ameliorate the deficiencies in selective attention observed in schizophrenics, but it remains unclear whether there are sex differences in this response to antipsychotic medication.

LI can be assessed in both rodents and humans, and LI paradigms are used to model the attentional deficits associated with schizophrenia in animals (Lubow, 2005). Repeated psychostimulant treatment, shown to induce a psychotic state in humans (Lichlyter et al., 2011), abolishes LI in rats (Moran et al., 1996; McAllister, 1997; Ruob et al., 1997). Administration of antipsychotics, including haloperidol (HAL; Feldon and Weiner, 1991; Dunn et al., 1993; Ruob et al., 1997; Weiner et al., 1997; Arad and Weiner, 2009), among others (Moran et al., 1996; Arad and Weiner, 2009), facilitate LI in rats. These antipsychotics also recover LI following a chronic psychostimulant treatment that abolishes LI (Moser et al., 2000), demonstrating that antipsychotics have similar effects on behavior in the LI paradigm in both rats and humans.

Experiments examining the effect of estrogens on behavior of rats in an LI paradigm have yielded contradictory results. Research from our lab and others indicates that high circulating levels of estrogens on the conditioning day of an LI paradigm, either during the proestrus phase of the estrous cycle or in response to estradiol (E2) replacement following ovariectomy, are associated with disrupted LI (Arad and Weiner, 2008; Nofrey et al., 2008; Quinlan et al., 2010). Correspondingly, low levels of circulating estrogens on the conditioning day of the same LI paradigm, either during the estrus phase of the estrous cycle or following ovariectomy, are associated with intact LI (Arad and Weiner, 2008; Nofrey et al., 2008; Quinlan et al., 2010). Recent research in humans used a paradigm that elicited LI in males, but not females (Vol’f et al., 2001; Kaplan and Lubow, 2011), which suggests that estrogens may also have detrimental effects on LI in humans. In contrast to these findings, it has also been shown that elimination of circulating estrogens via ovariectomy disrupts LI, and estrogen replacement following ovariectomy eliminates this deficit in LI (Arad and Weiner, 2009, 2010a,b). This would suggest that estrogens facilitate, not disrupt, selective attention. Research is needed to clarify the effect of estrogens on behavior observed in the LI paradigm.

The current experiments investigated the effects of E2 on selective attention, and the combined effects of both E2 and HAL on selective attention. In the first experiment, the behavior of males and ovariectomized (OVX) females receiving no E2 replacement, a low chronic E2 replacement, or a high phasic E2 replacement, were compared in a conditioned emotional response LI paradigm. This experiment also administered two doses of the antipsychotic, HAL (0.05 and 0.1 mg/kg), to determine, in a dose dependent manner, if the effects of HAL on LI varied based on sex or E2 replacement. In the first experiment, the aim was to use E2 replacement doses that mimic levels of E2 observed during the diestrus and proestrus phases of the estrous cycle. To ensure that E2 replacement doses achieved the desired levels of circulating E2, a second study was conducted. That study examined plasma levels of E2 in OVX rats administered the same E2 regimens as study one to quantify the plasma concentration of E2 over time following the E2 replacement regimes used here.

## Methods

### Subjects and surgeries

Subjects included 58 male and 179 OVX female Sprague–Dawley rats weighing approximately 260 g or 240 g, respectively, at the beginning of the experiment (Charles River Laboratories, St Constant, QC). Rats were housed in shoebox cages in a colony room maintained on a reversed 12:12 h light/dark cycle (lights off at 08:00 h) at approximately 21°C. Prior to surgery the animals were housed in pairs (but separated by sex), and following surgery animals were individually housed. All animals were handled daily, except during recovery from surgery. Food was available ad libitum throughout the experiment. Water was available ad libitum until a day before the start of the experiment, at which time water bottles were removed from cages and replaced for 30 min 2 h after the end of the daily experimental session. All animal handling and testing procedures were approved by the Animal Research Ethics Committee (AREC) of Concordia University, and were in accordance with guidelines established by the Canadian Council on Animal Care.

Approximately 4–7 days after arrival all animals underwent surgery. Females were OVX bilaterally through a lumbar incision, and males received sham surgeries, under isofluorane gas anesthetic (4% for induction, 2% for maintenance) using aseptic procedures. Post-surgical care included administration of the analgesic Anafen (0.1 ml, subcutaneous (SC)), the antibiotic penicillin (0.1 ml, IM), and physiological saline to prevent dehydration (3 ml, SC). All rats received a week-long recovery period prior to the start of the experiments.

### Drug and hormone treatments

Female rats were assigned to one of three hormone treatment groups: no E2 replacement, low chronic E2 replacement, or high phasic E2 replacement. At the time of OVX surgery rats in both the low and high E2 groups were implanted subcutaneously with a silastic capsule containing 5% 17-beta E2 (Sigma-Aldrich, St. Louis, MO) in cholesterol (Sigma). These capsules have been reported to produce a consistent serum concentration of E2 of ~20 pg/ml (Mannino et al., 2005), which is within the range observed in the diestrus phase of the estrous cycle (Overpeck et al., 1978). Rats in the high phasic E2 treatment group received a SC injection of E2 (10 µg/kg, SC) dissolved in sesame oil every 4th day to mimic levels of E2 observed during the proestrus phase of the cycle. All other rats received oil vehicle injections at this time. Injections of E2 or vehicle were administered after the session in the operant chambers on day 3 and 7 of the experiment. E2 was administered on day 7 so rats would be exposed to E2 approximately 16 h prior to the conditioning session on day 8 of the experiment.

HAL (diluted in 0.9% saline; Sandoz Inc, QC, Canada) was administered at 0, 0.05 or 0.1 mg/kg, IP. Doses were selected following a dose-response pilot study. A pilot study initially used a range of doses up to 0.2 mg/kg IP of HAL, corresponding to typical doses administered to males (Moser et al., 2000) and females (Arad and Weiner, 2009). However, this higher dose induced sluggishness and torpor in the high E2 female rats, evidenced by a lack of voluntary movement and diminished reaction to the footshock. Consequently, the HAL treatments were restricted to 0.05 mg/kg and 0.1 mg/kg. HAL injections were administered to the rats on the morning of day 8 of the experiment ~45 min before the beginning of the conditioning session.

### Latent inhibition

Modular operant test chambers (25 cm wide × 30 cm long × 30 cm high) contained within sound-attenuating isolation units were used for all behavioral training and testing (Coulbourne Instruments, Allentown, PA). Each chamber was equipped with a center house light (2.8 W) 27 cm above the grid floor in the center of the left hand wall of the box. A speaker was located just below the house light, and the grid floor was connected to a shock module. A water lickometer was located across from the house light approximately 8 cm above the floor. Every time an animal licked the water bottle positioned behind the lickometer an infrared beam was interrupted; the number of infrared beam interruptions was used as the measure of drinking behavior. Conditioning chambers and data acquisition were controlled by a desktop personal computer running Graphic State Notation software (Coulbourne Instruments, Allentown, PA).

Water restriction began the afternoon prior to the beginning of behavioral testing, at which point water bottles were removed from the home cages. The following morning rats were placed in the operant test chambers and allowed access to the water for 20 min; this habituation continued for the first 6 days of the experiment to guarantee that the rats learned to drink water in the chamber. Rats that did not learn to drink water in the operant boxes sufficiently (100 licks per session) were omitted from the study. The number of licks per session was recorded as baseline drinking behavior. Following habituation, on day 7 of the experiment, water bottles were removed from the operant chambers. Half of the animals from each group were presented with 40, 5 sec, 2.5 kHz tones at a volume of ~65 decibels (Preexposed group, PE). The 40 tone presentations were presented at 10–50 sec variable intervals over a 22.5 min session in the operant chambers. The other half of the animals were placed in the test chambers for an equivalent amount of time but were not presented with the tone (Not Preexposed group, NPE). On day 8 all rats underwent a conditioning session where they were subjected to two pairings of the 5 sec tone directly followed by a 0.5 mA foot shock for a duration of 1 sec. The intershock interval was 5 min. On the 9th day the water bottles were returned to the boxes and animals were re-habituated to drinking in the chambers.

Testing occurred on the 10th day when water bottles were present in the operant boxes. After the rats made 100 licks from the water bottle the tone turned on and remained on until the rat made 20 additional licks or until 5 min elapsed. This allowed for measurement of drinking behavior both with and without the tone. In this paradigm it is hypothesized that since the tone was previously paired with the footshock, subsequent presentation of the tone should inhibit drinking. The inhibition of drinking behavior is expected to be greater in rats in the NPE group, who were only presented with the tone paired with a footshock. Conversely, the PE group who heard the tone 40 times without any consequence should not associate the tone and footshock as strongly, and their drinking should be less inhibited. This paradigm is thought to indirectly measure freezing behavior, since the decrease in drinking following the onset of the tone is typically the result of the rat freezing, a characteristic fear response (Sotty et al., 1996). LI is considered to have occurred when the rats in the PE group make 20 licks with the tone on faster than rats in the NPE group.

### E2 plasma level assessment

A separate study, using different animals, was conducted to assess the plasma level accuracy of the E2 treatments used in the behavioral experiment. To examine the efficacy of the silastic capsule alone, E2 plasma concentrations were examined for 1 month following its implantation. This experiment included five female Sprague–Dawley rats that were OVX by a lumbar incision in the exact manner as described for the previous experiment (see Section Drug and Hormone Treatments). 1 week post ovariectomy, rats were implanted with a silastic capsule containing 5% E2 as described in the previous study. Blood was collected from the tail vein of these rats 1 week following ovariectomy, prior to E2 implant, and again at 1, 2, 3, and 4 weeks following capsule implantation.

In addition, to examine the plasma level effects of an acute E2 injection, 17 female Sprague–Dawley rats were OVX by a lumbar incision and implanted with an E2 containing silastic capsule as described previously. 10 days following the surgery all rats were administered a 10 µg/kg IP injection of E2 dissolved in sesame oil (see Section Drug and Hormone Treatments). E2 was assayed in sera from rats at six time points across a 24 h period; any one rat was only used for two time points for ethical reasons. Blood was collected from the tail vein of five rats just prior to the injection (baseline), and then again 4 h following the injection. Blood was collected from six different rats at 8 h and again at 12 h following the injection. Finally, blood was collected from the final six rats at 16 h and again at 20 h following injection.

Blood was collected in ice-cold vials and immediately centrifuged. Plasma was stored at −20°C until being assayed for E2 using a commercially available ELISA kit (Immuno-Biological Laboratories Inc., Minneapolis, MI). The assay antibodies have 100% cross-reactivity with E2 and 0.2% and 0.05% cross-reactivity with estrone and estriol, respectively. The range of the assay is between 0 and 2000 pg/ml and the reported inter-assay variation is 7–9%.

### Statistical analyses

In an LI protocol the suppression ratios in the PE and NPE groups are compared to determine if LI has occurred in the PE group. For each rat, a suppression ratio was calculated as a measure of LI. This was measured as the time to complete licks 81–100 [A—without the tone] divided by the sum of the time to complete licks 81–100 and licks 101–120 [B—with the tone] (A/A + B). Here, a suppression ratio of 0.5 indicates no suppression of licking during the tone (i.e., no conditioned response to the tone), while a suppression ratio of 0.003 indicates full suppression of licking in the presence of the tone. Suppression ratios for the rats in each group were averaged; LI is considered to have occurred when the rats in the PE group have significantly higher suppression ratio than that in those in the NPE group.

Data from the males and females were analyzed separately, as hormone treatment level was a factor in the analyses for the females but not for the males. Suppression ratios from the males were analyzed using a 2 × 3 between subjects ANOVA, and suppression ratios for the females were analyzed using a 2 × 3 × 3 between subjects ANOVA. Additionally, planned orthogonal contrasts were run on all of the data, comparing the PE and NPE groups in each condition to determine if LI occurred. T-tests were used for all orthogonal contrasts, and Cohen’s d effect size calculations were run for each t-test. Data from study 2, examining circulating levels of E2 in OVX females with E2 replacement were not statistically analyzed, as these data were meant to be descriptive.

## Results

### Latent inhibition

**Figure 1:**
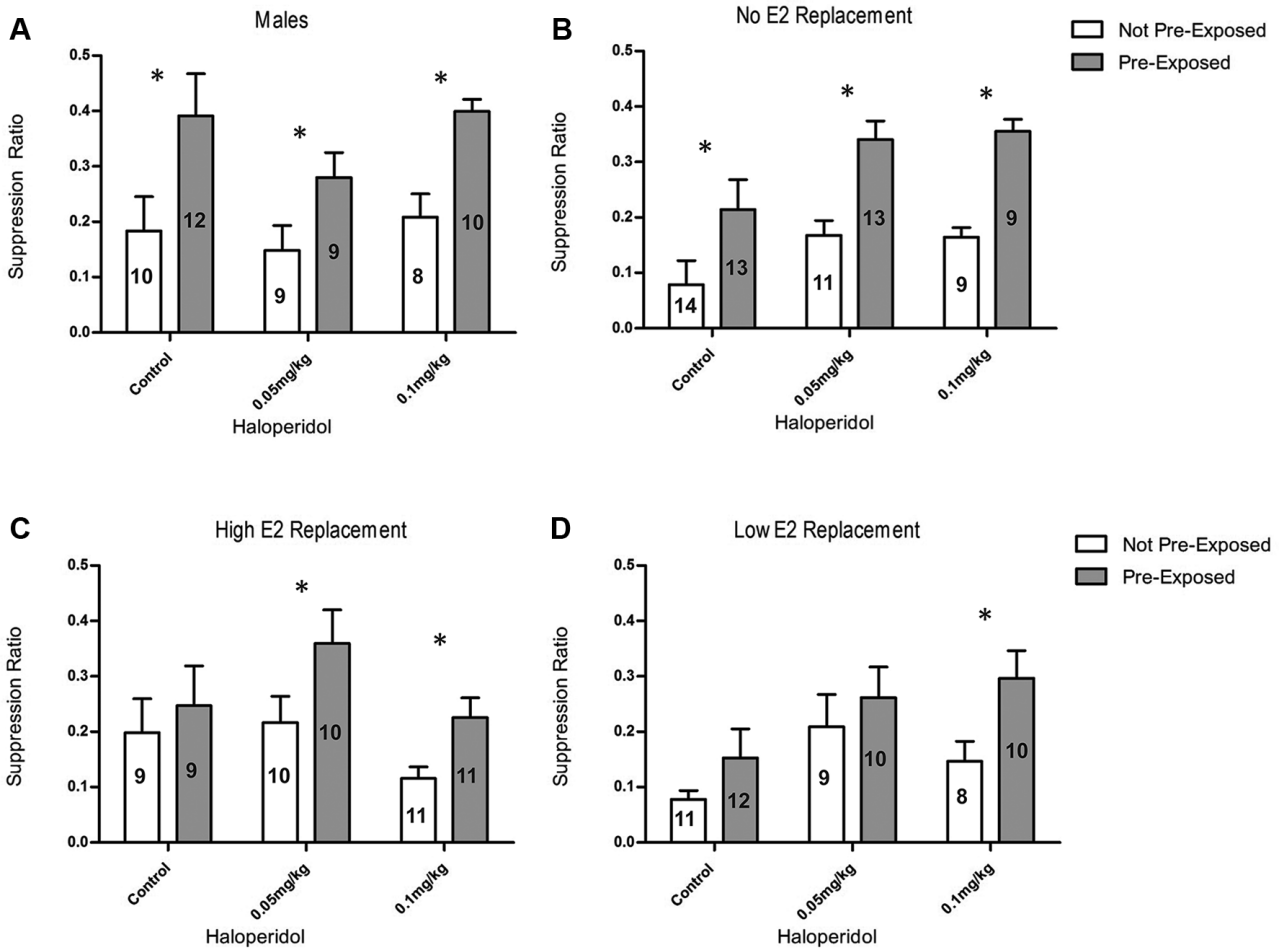
**(A)** Suppression ratios for males; LI was observed in rats treated with 0.05 and 0.1 mg/kg HAL. **(B)** Suppression ratios for OVX females receiving no E2 replacement; LI was observed in rats treated with vehicle, 0.05 mg/kg, and 0.1 mg/kg HAL. **(C)** Suppression ratios for ovariectomized (OVX) females receiving high E2 replacement; LI was observed in rats treated with 0.05 mg/kg and 0.1 mg/kg HAL. **(D)** Suppression ratios for OVX females receiving low E2 replacement: LI was observed in rats treated with the 0.1 mg/kg HAL. The error bars represent standard error of the mean, the *n* for each group is depicted on the bar representing that group. (* = *p* < 0.05).

Male rats exhibited LI regardless of HAL treatment (Figure 1A). A 2 × 3 ANOVA, with main factor of exposure (PE vs NPE) and HAL dose (vehicle, 0.05 mg/kg, 0.1 mg/kg), was used to analyze data from the male rats. This ANOVA revealed a significant main effect of exposure, *F*(1, 52) = 19.41, *p* < 0.001. This main effect was due to significantly higher suppression ratios in the PE group, compared to the NPE group, indicating that LI occurred. Orthogonal contrasts demonstrated that the PE rats had significantly higher suppression ratios than the NPE rats in the vehicle group (*t*(20) = 2.42, *p* = 0.023, *d* = 1.11), the 0.05 mg/kg HAL group (*t*(16) = 3.13, *p* = 0.004, *d* = 1.66), and the 0.1 mg/kg HAL group (*t*(16) = 3.77, *p* = 0.002, *d* = 1.88). This shows that male rats developed LI regardless of the dose of HAL.

A 2 × 3 × 3 ANOVA, with main factors of exposure (PE vs NPE), E2 replacement (no E2, low E2, and high E2), and HAL dose (vehicle, 0.05 mg/kg, 0.1 mg/kg), was used to analyze data from the female rats. There was a main effect of exposure, *F*(1, 173) = 27.37, *p* < 0.001, a main effect of HAL treatment, *F*(2, 173) = 6.12, *p* = 0.003), and an interaction between exposure and HAL treatment, *F*(4, 173) = 2.74, *p* = 0.030. Orthogonal contrasts demonstrated that OVX females receiving no E2 replacement in the PE group had significantly higher suppression ratios than NPE group in the vehicle condition (*t*(25) = 2.32, *p* = 0.029, *d* = 0.93), the 0.05 mg/kg HAL condition (*t*(22) = 2.45, *p* = 0.023, *d* = 1.04), and the 0.1 mg/kg condition (*t*(16) = 2.92, *p* = 0.010, *d* = 1.46; Figure 1B). These OVX females showed a similar pattern of results to the male rats, exhibiting LI regardless of the dose of HAL. Orthogonal contrasts demonstrated that OVX rats with either high or low E2 replacement (Figures 1C,D, respectively) did not develop LI when administered a vehicle injection (*d* = 0.33 and *d* = 0.62, respectively). At the 0.05 mg/kg dose of HAL, rats in the high E2 group exhibited LI, that is, the PE rats exhibited significantly higher suppression ratios than the NPE rats (*t*(10) = 2.35, *p* = 0.03, *d* = 1.11). In contrast, rats receiving low E2 replacement and the 0.05 mg/kg dose of HAL did not express LI insofar as there was no difference in suppression ratios between PE and NPE groups (*d* = 0.71). Finally, the 0.1 mg/kg dose of HAL recovered LI in rats receiving both the high and low E2 replacement (*t*(11) = 2.69, *p* = 0.02, *d* = 1.20, and *t*(10) = 2.49, *p* = 0.02, *d* = 1.21, respectively).

### E2 plasma levels

**Figure 2:**
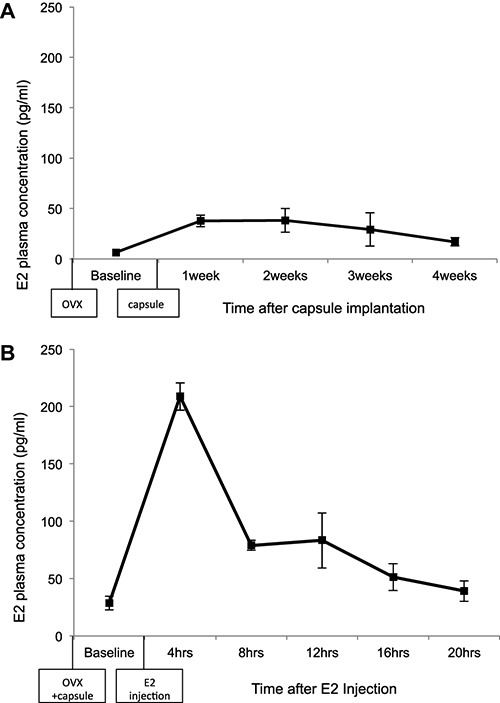
**Estradiol (E2) plasma levels following**: **(A)** ovariectomy and implantation of a silastic capsule containing 5% 17-beta E2 and cholesterol. **(B)** Ovariectomy, implantation of a silastic capsule, and a 10 µg/kg SC injection of E2, dissolved in sesame oil. The error bars represent standard error of the mean.

The plasma concentrations of E2 following implantation of the silastic capsule were determined (Figure 2A). Data are expressed as mean ± standard error of the mean. Female rats had relatively low levels of E2 after ovariectomy, prior to implantation of the capsule (6.51 ± 1.38 pg/ml). There was an increase in the plasma concentration of E2 at week 1 (37.54 ± 5.67 pg/ml) and week 2 (38.25 ± 11.72 pg/ml) following capsule implantation. Plasma E2 levels slowly decreased at week 3 (29.41 ± 16.58 pg/ml) and week 4 (17.09 ± 4.01 pg/ml). These plasma concentrations are in the range of the average plasma level of E2 during diestrus in the rat (Overpeck et al., 1978).

A second experiment examined the plasma concentration of E2 in response to a silastic capsule implant paired with a 10 µg/kg injection of E2 (Figure 2B). Rats had similar E2 plasma concentrations following capsule implantation as was observed in the previous experiment (28.66 ± 5.91 pg/ml), confirming the efficacy of the capsules. 4 h following the E2 injection the average plasma level of E2 increased markedly to 208.79 ± 12.03 pg/ml. The plasma concentration of E2 had decreased to 79.00 ± 4.30 pg/ml 8 h following the injection, and was maintained close to that level 12 h following injection (83.26 ± 24.08 pg/ml). 16 h after the injection plasma concentrations of E2 dropped to 51.30 ± 11.68 pg/ml, and 20 h following the injection plasma levels were down to 39.18 ± 8.97 pg/ml (Figure 2B). The average plasma concentration across the 20 h period following E2 injection is in the range of plasma levels observed during the proestrus phase of the cycle in the rat (Overpeck et al., 1978).

## Discussion

These experiments demonstrate that physiologically relevant levels of E2 disrupt LI, and this disruption can be reversed by an acute injection of HAL administered prior to the conditioning session. Furthermore, the lower dose of HAL (0.05 mg/kg) was sufficient to restore LI in the high E2 replacement rats, but not in those with low E2 replacement. This indicates that estrogens may facilitate the effects of HAL, as HAL is more effective in females with high levels of E2. Females are more responsive to antipsychotics than males (Seeman, 2004), and these results suggest that estrogens contribute to this sex difference differing responses to HAL in males and females in an LI paradigm. These experiments also show that the dose and method of E2 capsule replacement used here results in sustained low levels of E2 similar to that observed during diestrus for up to 4 weeks. Furthermore, the single injection of E2 shows a peak plasma concentration within 4 h following injection, returning to baseline levels within 20 h mimicking estrogen levels occurring during proestrus (Overpeck et al., 1978).

### E2 and latent inhibition

The results presented here are in accordance with previous research from this lab indicating that estrogens disrupt LI in females under conditions where LI is observed in males and in OVX female rats (Nofrey et al., 2008; Quinlan et al., 2010). When OVX rats are administered E2 replacement alone, there is no difference between suppression ratios in the PE and NPE groups, indicating that LI did not occur (Figure 1). We did not observe a difference between PE and NPE females due to low suppression ratios in the PE group, suggesting that the E2 treated females are unable to ignore the tone that should have been rendered irrelevant through multiple non-reinforced presentations. In contrast both male rats and OVX female rats given no E2 replacement have significantly higher suppression ratios in the PE group compared to the NPE group, indicating that LI did occur. Taken together these findings demonstrate that estrogen has detrimental effects on selective attention, impairing the ability to disregard irrelevant stimuli in the environment.

In contrast to these findings there is a body of research indicating that ovariectomy induces deficits in LI while E2 facilitates LI (Arad and Weiner, 2009, 2010a,b). The reason for these contradictory results is unclear, although there are methodological differences which likely contributed. In the conditioned emotional response LI paradigm employed by others (Arad and Weiner, 2009, 2010a,b) they use a 10 sec, 80 dB tone during pre-exposure and conditioning compared to the 5 sec 65 dB tone used in our experiments. Therefore their stimuli is much more salient, since it is louder and longer, which could impact whether the stimuli is rendered irrelevant during the preexposure phase, and the association formed during the conditioning phase. Additionally, our high E2 replacement dose is much lower than the E2 dose used by other researchers in the field. In this study, efforts were made to use an E2 replacement regime that mimicked the estrous cycle of the rats. Study two demonstrates that plasma levels of E2 following capsule implantation, and capsule implantation combined with an acute injection E2, are within the range observed during the diestrus and proestrus phases of the cycle, respectively (Figure 2; Overpeck et al., 1978), so the results of this experiment should mimic the effects of endogenous estrogens. This difference in the E2 replacement doses may provide a partial explanation for the contradictory findings on the effects of E2 on selective attention. In addition, LI as employed here is affected by many variables including level of conditioning to the tone-footshock pairing as well as general anxiety levels. It is possible that some of the effects observed here could be attributed to these as well as other variables unrelated to selective attention.

### Haloperidol and LI

It is well established that HAL can facilitate LI in male rats (Moser et al., 2000), and more recently research has demonstrated that this is also true for female rats (Arad and Weiner, 2009). The findings of the current experiments correspond to this, as the highest dose of HAL restores LI in females receiving both low and high E2 replacement. More interestingly these results indicate that E2 actually enhances the effects of HAL on LI, as the lower dose of HAL was sufficient to restore LI in the high E2 replacement group, but not in the low E2 replacement group. In these experiments, and others (Arad and Weiner, 2008; Nofrey et al., 2008; Quinlan et al., 2010), elevated levels of circulating estrogens are detrimental to LI in female rats, but these findings indicate that E2 replacement also facilitates the effects of HAL treatment in facilitating LI. There is evidence that this might be the case in humans as well. Sex differences in LI are observed in humans, where males exhibit LI under conditions where females do not (Vol’f et al., 2001; Kaplan and Lubow, 2011), and the peak of estrogens in the menstrual cycle is correlated with distractibility and an inability to disengage from irrelevant stimuli (Beaudoin and Marrocco, 2005). Additionally, research in humans indicates that antipsychotic treatments are more effective in females, as schizophrenic women require significantly lower doses of antipsychotic drugs to alleviate their symptoms than men (Usall et al., 2007). Furthermore E2 administered in conjunction with antipsychotic treatment results in better treatment outocomes in schizophrenia (Akhondzadeh et al., 2003; Seeman, 2004).

This study did not examine the neurobiological underpinnings of the behaviors observed in the LI paradigm. However, previous research has implicated dopaminergic transmission in various nuclei of the mesocorticolimbic system in LI. In the prefrontal cortex (PFC), lesions and local infusion of a dopamine antagonist enhance LI (Broersen et al., 1996; George et al., 2010). Lesions of the nucleus accumbens (NAc) abolish LI (Weiner et al., 1997), and dopaminergic activity in the anterior striatum (STR) is positively correlated with behavior in an LI task (Jeanblanc et al., 2003). Interestingly, estrogens have been shown to alter dopamine transmission in these nuclei of the mesocorticolimbic system. Specifically, estrogens increase dopamine levels and dopamine 2 receptor (D2R) density in the NAc (Thompson and Moss, 1994; Le Saux et al., 2006), increase dopamine activity and D2R density in the STR (Becker and Rudick, 1999; Landry et al., 2002), and increase dendritic spine and dopamine one receptor density in the PFC (Lévesque and Di Paolo, 1989; Wallace et al., 2006). Estrogen-induced changes in dopamine transmission in the mesocorticolimbic system could be partially responsible for the detrimental effects of estrogens on behavior in the LI paradigm. Additionally, HAL exerts its effects by antagonizing D2Rs, and E2 replacement increases in D2R density in the STR and NAc (Landry et al., 2002; Le Saux et al., 2006). Therefore, E2 replacement would increase binding sites for HAL, providing one possible explanation for estrogens’ facilitatory effects on this antipsychotic. Future research should investigate these hypotheses to determine whether estrogen-induced changes in dopaminergic transmission in the mesocorticolimbic system are responsible for the E2-induced reductions in selective attention, and increased response to HAL treatment, observed in this study.

## Conclusion

E2 replacement administered to OVX females abolishes LI under conditions where it is observed in males, but despite these detrimental effects on LI, E2 facilitates HAL to enhance LI. These experiments indicate that estrogens are, in part, responsible for sex differences in LI and the response to HAL. Further research is needed to extend these findings from HAL to other antipsychotic treatments, and to elucidate the specific neurobiological mechanisms responsible for estrogens effects on LI and the response to antipsychotic treatments.

## Author Contributions

Anne Almey conducted the research with assistance from Nada Hafez and Arne Hantson. The manuscript was written by Anne Almey and Wayne Brake supervised the project and edited the manuscript.

## Conflict of interest statement

The authors declare that the research was conducted in the absence of any commercial or financial relationships that could be construed as a potential conflict of interest.

## References

[B1] Akhondzadeh S. Nejatisafaa A. A. Aminia H. Mohammadia M. R. Larijanic B. Kashanid L. (2003). Adjunctive estrogen treatment in women with chronic schizophrenia: a double-blind, randomized, and placebo-controlled trial. Prog. Neuropsychopharmacol. Biol. Psychiatry 27, 1007–1012 10.1016/s0278-5846(03)00161-1 14499318

[B2] Arad M. Weiner I. (2008). Fluctuation of latent inhibition along the estrous cycle in the rat: modeling the cyclicity of symptoms in schizophrenic women? Psychoneuroendocrinology 33, 1401–1410 10.1016/j.psyneuen.2008.08.001 18819755

[B3] Arad M. Weiner I. (2009). Disruption of latent inhibition induced by ovariectomy can be reversed by estradiol and clozapine as well as by co-administration of haloperidol with estradiol but not by haloperidol alone. Psychopharmacology (Berl) 206, 731–740 10.1007/s00213-009-1464-0 19169876

[B4] Arad M. Weiner I. (2010a). Sex-dependent antipsychotic capacity of 17β-Estradiol in the latent inhibition model: a typical antipsychotic drug in both sexes, atypical antipsychotic drug in males. Neuropsychopharmacology 35, 2179–2192 10.1038/npp.2010.89 20613719 PMC3055319

[B5] Arad M. Weiner I. (2010b). Contrasting effects of increased and decreased dopamine transmission on latent inhibition in ovariectomized rats and their modulation by 17beta-estradiol: an animal model of menopausal psychosis? Neuropsychopharmacology 35, 1570–1582 10.1038/npp.2010.28 20237462 PMC3055453

[B6] Beaudoin J. Marrocco R. (2005). Attentional validity effect across the human menstrual cycle varies with basal temperature changes. Behav. Brain Res. 158, 23–29 15680191 10.1016/j.bbr.2004.08.005

[B7] Becker J. B. Rudick C. N. (1999). Rapid effects of estrogen or progesterone on the amphetamine-induced increase in striatal dopamine are enhanced by estrogen priming: a microdialysis study. Pharmacol. Biochem. Behav. 64, 53–57 10.1016/s0091-3057(99)00091-x 10494997

[B8] Brébion G. Smith M. J. Gorman J. M. Amador X. (1996). Reality monitoring failure in schizophrenia: the role of selective attention. Schizophr. Res. 22, 173–180 10.1016/s0920-9964(96)00054-0 8958602

[B9] Broersen L. M. Heinsbroek R. P. de Bruin J. P. Olivier B. Broersen L. M. Heinsbroek R. (1996). Effects of local application of dopaminergic drugs into the medial prefrontal cortex of rats on latent inhibition. Biol. Psychiatry 40, 1083–1090 10.1016/s0006-3223(95)00595-1 8931910

[B10] Dunn L. A. Atwater G. E. Kilts C. D. (1993). Effects of antipsychotic drugs on latent inhibition: sensitivity and specificity of an animal behavioral model of clinical drug action. Psychopharmacology (Berl) 112, 315–323 10.1007/bf02244927 7871036

[B11] Escobar M. Oberling P. Miller R. R. (2002). Associative deficit accounts of disrupted latent inhibition and blocking in schizophrenia. Neurosci. Biobehav. Rev. 26, 203–216 10.1016/s0149-7634(01)00067-7 11856559

[B12] Feldon J. Weiner I. (1991). The latent inhibition model of schizophrenic attention disorder. Haloperidol and sulpiride enhance rats’ ability to ignore irrelevant stimuli. Biol. Psychiatry 29, 635–646 10.1016/0006-3223(91)90133-7 2054435

[B13] George D. N. Duffaud A. M. Pothuizen H. H. Haddon J. E. Killcross S. (2010). Lesions to the ventral, but not the dorsal, medial prefrontal cortex enhance latent inhibition. Eur. J. Neurosci. 31, 1474–1482 10.1111/j.1460-9568.2010.07178.x 20384772

[B14] Gray J. A. (1998). Integrating schizophrenia. Schizophr. Bull. 24, 249–266 10.1093/oxfordjournals.schbul.a033324 9613624

[B15] Häfner H. (2003). Gender differences in schizophrenia. Psychoneuroendocrinology 28(Suppl. 2), 17–54 10.1016/S0306-4530(02)00125-7 12650680

[B16] Häfner H. Riecher-Rössler A. Maurer K. Fätkenheuer B. Löffler W. (1992). First onset and early symptomatology of schizophrenia: a chapter of epidemiological and neurobiological research into age and sex differences. Eur. Arch. Psychiatry Clin. Neurosci. 242, 109–118 10.1007/bf02191557 1486099

[B17] Jeanblanc J. Hoeltzel A. Louilot A. (2003). Differential involvement of dopamine in the anterior and posterior parts of the dorsal striatum in latent inhibition. Neuroscience 118, 233–241 10.1016/s0306-4522(02)00823-0 12676153

[B18] Kaplan O. Lubow R. E. (2011). Ignoring irrelevant stimuli in latent inhibition and stroop paradigms: the effects of schizotypy and gender. Psychiatry Res. 186, 40–45 10.1016/j.psychres.2010.07.025 20797796

[B19] Kendell R. E. Chalmers J. C. Platz C. (1987). Epidemiology of puerperal psychoses. Br. J. Psychiatry 150, 662–673 10.1192/bjp.150.5.662 3651704

[B21] Kulkarni J. Gurvich C. Gilbert H. Mehmedbegovic F. Mu L. Marston N. (2008). Hormone modulation: a novel therapeutic approach for women with severe mental illness. Aust. N Z J. Psychiatry 42, 83–88 10.1080/00048670701732715 18058448

[B22] Landry M. Lévesque D. Di Paolo T. (2002). Estrogenic properties of raloxifene, but not tamoxifen, on D2 and D3 dopamine receptors in the rat forebrain. Neuroendocrinology 76, 214–222 10.1159/000065951 12411738

[B23] Le Saux M. Morissette M. Di Paolo T. (2006). ERbeta mediates the estradiol increase of D2 receptors in rat striatum and nucleus accumbens. Neuropharmacology 50, 451–457 10.1016/j.neuropharm.2005.10.004 16309717

[B24] Lévesque D. Di Paolo T. (1989). Chronic estradiol treatment increases ovariectomized rat striatal D-1 dopamine receptors. Life Sci. 45, 1813–1820 10.1016/0024-3205(89)90522-5 2531825

[B25] Lichlyter B. Purdon S. Tibbo P. (2011). Predictors of psychosis severity in individuals with primary stimulant addictions. Addict. Behav. 36, 137–139 10.1016/j.addbeh.2010.08.019 20850224

[B26] Lubow R. E. (1989). Latent Inhibition and Conditioned Attention Theory. New York: Cambridge University Press, 1–8

[B27] Lubow R. E. (2005). Construct validity of the animal latent inhibition model of selective attention deficits in schizophrenia. Schizophr. Bull. 31, 139–153 10.1093/schbul/sbi005 15888432

[B28] Lubow R. E. Kaplan O. Abramovich P. Rudnick A. Laor N. (2000). Visual search in schizophrenia: latent inhibition and novel pop-out effects. Schizophr. Res. 45, 145–156 10.1016/s0920-9964(99)00188-7 10978882

[B29] Mannino C. A. South S. M. Inturrisi C. E. Quinones-Jenab V. (2005). Pharmacokinetics and effects of 17beta-estradiol and progesterone implants in ovariectomized rats. J. Pain 6, 809–816 10.1016/j.jpain.2005.07.007 16326369

[B45] Matevosyan N. R. (2011). Pregnancy and postpartum specifics in women with schizophrenia: a meta-study. Arch. Gynecol. Obstet. 283, 141–147 10.1007/s00404-010-1706-8 20931211

[B30] McAllister K. H. (1997). A single administration of d-amphetamine prior to stimulus pre-exposure and conditioning attenuates latent inhibition. Psychopharmacology (Berl) 130, 79–84 10.1007/s002130050213 9106903

[B31] Moran P. M. Fischer T. R. Hitchcock J. M. Moser P. C. (1996). Effects of clozapine on latent inhibition in the rat. Behav. Pharmacol. 7, 42–48 10.1097/00008877-199601000-00003 11224392

[B32] Moser P. C. Hitchcock J. M. Lister S. Moran P. M. (2000). The pharmacology of latent inhibition as an animal model of schizophrenia. Brain Res. Brain Res. Rev. 33, 275–307 10.1016/s0165-0173(00)00026-6 11011070

[B33] Nofrey B. S. Ben-Shahar O. M. Brake W. G. (2008). Estrogen abolishes latent inhibition in ovariectomized female rats. Brain Cogn. 66, 156–160 10.1016/j.bandc.2007.06.003 17693005

[B34] Overpeck J. G. Colson S. H. Hohmann J. R. Applestine M. S. Reilly J. F. (1978). Concentrations of circulating steroids in normal prepubertal and adult male and female humans, chimpanzees, rhesus monkeys, rats, mice, and hamsters: a literature survey. J. Toxicol. Environ. Health 4, 785–803 10.1080/15287397809529700 104044

[B35] Quinlan M. G. Duncan A. Loiselle C. Graffe N. Brake W. G. (2010). Latent inhibition is affected by phase of estrous cycle in female rats. Brain Cogn. 74, 244–248 10.1016/j.bandc.2010.08.003 20817338

[B36] Ruob C. Elsner J. Weiner I. Feldon J. (1997). Amphetamine-induced disruption and haloperidol-induced potentiation of latent inhibition depend on the nature of the stimulus. Behav. Brain Res. 88, 35–41 10.1016/s0166-4328(97)02305-x 9401706

[B37] Schmidt-Hansen M. Killcross A. S. Honey R. C. (2009). Latent inhibition, learned irrelevance, and schizotypy: assessing their relationship. Cogn. Neuropsychiatry 14, 11–29 10.1080/13546800802664539 19214840

[B38] Seeman M. V. (2004). Gender differences in the prescribing of antipsychotic drugs. Am. J. Psychiatry 161, 1324–1333 10.1176/appi.ajp.161.8.1324 15285956

[B39] Sotty F. Sandner G. Gosselin O. (1996). Latent inhibition in conditioned emotional response: c-fos immunolabelling evidence for brain areas involved in the rat. Brain Res. 737, 243–254 10.1016/0006-8993(96)00737-8 8930372

[B40] Thompson T. L. Moss R. L. (1994). Estrogen regulation of dopamine release in the nucleus accumbens: genomic- and nongenomic-mediated effects. J. Neurochem. 62, 1750–1756 10.1046/j.1471-4159.1994.62051750.x 8158125

[B41] Usall J. Suarez D. Haro J. M. (2007). Gender differences in response to antipsychotic treatment in outpatients with schizophrenia. Psychiatry Res. 153, 225–231 10.1016/j.psychres.2006.09.016 17681611

[B42] Vol’f N. V. Razumnikova O. M. Vasil’ev O. V. (2001). Differences in latent inhibition between men and women under conditions of nonverbal visually-spatial masking. Zh. Vyssh. Nerv. Deiat. Im. I P Pavlova 51, 558–562 11764514

[B43] Wallace M. Luine V. Arellanos A. Frankfurt M. (2006). Ovariectomized rats show decreased recognition memory and spine density in the hippocampus and prefrontal cortex. Brain Res. 1126, 176–182 10.1016/j.brainres.2006.07.064 16934233

[B44] Weiner I. Shadach E. Barkai R. Feldon J. (1997). Haloperidol- and clozapine-induced enhancement of latent inhibition with extended conditioning: implications for the mechanism of action of neuroleptic drugs. Neuropsychopharmacology 16, 42–50 10.1016/s0893-133x(96)00145-5 8981387

